# Pallial patterning and the origin of the isocortex

**DOI:** 10.3389/fnins.2015.00377

**Published:** 2015-10-14

**Authors:** Juan F. Montiel, Francisco Aboitiz

**Affiliations:** ^1^Facultad de Medicina, Centro de Investigación Biomédica, Universidad Diego PortalesSantiago, Chile; ^2^Medical Research Council Functional Genomics Unit, Department of Physiology, Anatomy and Genetics, University of OxfordOxford, UK; ^3^Departamento de Psiquiatría, Escuela de Medicina, and Centro Interdisciplinario de Neurociencia, Pontificia Universidad Católica de ChileSantiago, Chile

**Keywords:** isocortical development, Pax6, Wnt, antihem, hem

## Abstract

Together with a complex variety of behavioral, physiological, morphological, and neurobiological innovations, mammals are characterized by the development of an extensive isocortex (also called neocortex) that is both laminated and radially organized, as opposed to the brain of birds and reptiles. In this article, we will advance a developmental hypothesis in which the mechanisms of evolutionary brain growth remain partly conserved across amniotes (mammals, reptiles and birds), all based on Pax6 signaling or related morphogens. Despite this conservatism, only in mammals there is an additional upregulation of dorsal and anterior signaling centers (the cortical hem and the anterior forebrain, respectively) that promoted a laminar and a columnar structure into the neocortex. It is possible that independently, some birds also developed an upregulated dorsal pallium.

## Introduction

According to a developmental perspective, brain homologies across vertebrates are supported by anatomical topographic correspondence and embryonic expression of homeobox and homeobox-like genes. However, species-specific pallial morphology and global gene expression patterns exhibit important dissimilarities at adult stages that would be explained by changes in the differential modulation of pallial developmental programs. In this article we elaborate further the hypothesis that the evolution of the mammalian isocortex required the modulation of conserved developmental patterning programs, providing a new phenotype to the dorsal proliferative compartments. Specifically, this was achieved by virtue of a strong upregulation of dorsal patterning centers like the hem, in combination with the expansion of Pax6 expression that generated a subventricular zone (SVZ) that amplified the proliferation of neuronal progenitors. A third regulating component is provided by the rostral patterning center (RPC), that secretes molecules of the FGF family. Thus, the orchestration and partial overlap of dorsal, lateral and anterior patterning centers gave rise to the laminated mammalian isocortex as an expansion of the dorsal pallial field. This process will be compared to the mechanisms of brain amplification in reptiles and birds, with whom there are both similarities (upregulation of Pax6 signals) and divergences (the dorsal and anterior patterning centers display little upregulation in reptiles and birds).

## The brains of amniotes

In order to provide the required background to understand the arguments that follow, we will briefly summarize some of the main differences in anatomical brain organization between different amniotes (reptiles, birds and mammals) (Aboitiz et al., 2002, 2003b; Aboitiz and Montiel, 2007b). First, although there are major differences in adult anatomical brain structure, in both mammals and sauropsids (i.e., reptiles and birds) the cerebral hemispheres or telencephali retain the major subdivisions that characterize all vertebrates, i.e., a dorsal component or pallium, receiving most sensory afferents via specialized thalamic nuclei (with the exception of olfaction, that reaches the olfactory cortex directly through the olfactory tract), and a ventral component or subpallium, including the basal ganglia which are involved mainly (but not exclusively) in motor functions. While the subpallium has remained somewhat more conservative in vertebrate evolution, dramatic changes in pallial structure have been observed in the different vertebrate groups, including amniotes.

### Pallial subdivisions

In mammals, the pallium has a relatively simple structure, with a laminar organization that spans the medial pallium (the hippocampus), the dosal pallium (the isocortex) and the lateral pallium (the olfactory cortex), but this laminar pattern is distorted as an aggregate of neurons in the ventral pallial components that contribute to the amygdalar complex. The isocortex is clearly distinguished from the lateral (olfactory cortex) and medial (hippocampal region) pallial derivatives by its conspicuous six-layered structure (as opposed to the three-layered organization of the latter), which in addition is organized into radial columns that derive embryologically from cells following the same radial glial trajectory and in majority are clonally-related (Rakic, 2009; Gao et al., 2014; Vasistha et al., 2014).

In reptiles, the dorsalmost and medial aspects of the pallium are very small and barely make up a primitive layered structure that nonetheless differs from that of the more basal amphibians (the sister group of amniotes) by the existence, in the former, of a limited radial migratory capacity during development, which generates a rudimentary cortex in the adult. On the other hand, in the lateral and ventral pallium, i.e., on the equatorial aspect of the hemisphere, adjacent to the more ventral subpallium, a relatively large structure bulges inside the ventricular cavity, which is termed the dorsal ventricular ridge (DVR). Here we will refer to the anterior component of the DVR, which receives most sensory inputs while its posterior component is agreed to correspond to some amygdalar and subpallial elements (Abellán et al., 2009). As said, the DVR capitalizes most sensory afferences coming from the midbrain to the thalamus (mainly auditory and visual; called collothalamic pathways), while some other sensory afferences that reach directly the thalamus, bypassing the mesencephalon, project to the dorsal and dorsomedial pallium (visual and somatosensory, termed lemnothalamic pathways) (Butler, 1995). In the reptilian lateral pallium, there is also a lateral or olfactory cortex.

In birds, the ancestral DVR has become severely hypertrophied, subdividing into (i) a nidopallium (originating from the ventral pallium and receiving visual and auditory mesencephalic sensory input) and (ii) a mesopallium (originating from the lateral pallium; Medina and Abellán, 2009). Other avian components have also increased in complexity, particularly (iii) the hyperpallium (derived from the dorsal pallium and receiving lemnothalamic visual and somatosensory input), and (iv) the arcopallium (posterior DVR of reptiles, derived from the ventral pallium and subpallium, and comparable to some parts of the mammalian amygdala). All these structures have a morphology that is radically different from that of the isocortex of mammals, as they show no evident signs of laminar or radial organization (although there are important similarities in sensory connectivity and internal circuitry (Jarvis et al., 2005, 2013; Wang et al., 2010; Karten, 2013; Ahumada-Galleguillos et al., 2015; Calabrese and Woolley, 2015). The medial pallium of reptiles and birds is somewhat more conservative, retaining a laminar structure, and is widely considered to be directly comparable to at least parts of the mammalian hippocampus (Striedter, 2015).

### Controversies about homology

Thus, at first sight the brains of sauropsids and mammals are radically different in their anatomical organization. Attempts to establish homologies between these structures have been plagued with controversy, starting with Holmgren's (Holmgren, 1922) early suggestion that the sauropsidian DVR corresponded to components of the amygdalar complex in mammals and the proposal by Ariëns Kappers et al. (1936) that the DVR was a component of the subpallial basal ganglia. The now classical works by Harvey Karten, and by André Parent in the 1960s, established that the DVR was in fact a pallial component, by virtue of its collothalamic sensory afferents (Karten, 1969) and the absence of AChE immunoreactivity, a well-recognized marker of the basal ganglia (Parent and Olivier, 1970). Karten analyzed the collothalamic auditory and visual afferents to the avian nidopallium, noticing a striking similarity between these and the auditory and the so-called extrastriate visual afferents to the mammalian isocortex (both of which are also collothalamic). On the other hand, the portion of the neocortex receiving lemnothalamic visual (striate visual cortex) and somatosensory afferents has sensory connections similar to those in the hyperpallium, a dorsal pallial derivative (Puelles et al., 2000; Aboitiz et al., 2003b; Nomura et al., 2013). Furthermore, recent evidence has appeared showing a similar microcircuitry and input-output organization in the avian nidopallium and the mammalian isocortex (Wang et al., 2010; Ahumada-Galleguillos et al., 2015; Calabrese and Woolley, 2015). Based on this notable evidence, Karten and his associates have strongly advocated for the hypothesis of homology between the circuits in the avian DVR and those in the collothalamic-receiving isocortex (auditory and secondary visual areas). On the other hand, lemnothalamic-receiving isocortical regions (somatosensory and primary visual areas) would correspond to the dorsal cortex or hyperpallium of birds and reptiles, respectively (Butler et al., 2011).

On the other hand, another line of interpretation followed more closely Holmgren's hypothesis of homology between the DVR and parts of the amygdalar complex of mammals (Aboitiz, 1992; Bruce and Neary, 1995; Striedter, 1997; Puelles et al., 2000; Aboitiz et al., 2003b; Montiel and Molnár, 2013). This line emphasized the expansion of the dorsal pallium (the reptilian dorsal cortex) as the main event in neocortical origins, and assumed that most of the neocortex, including that receiving collothalamic afferents, was of dorsal pallial origin. This perspective received strong support from developmental tracing studies using neural and genetic markers that firmly established a ventral and lateral pallial origin for the sauropsidian DVR (recall, we are referring here to the anterior DVR), as opposed to the isocortex that derives from the dorsal pallium. Nonetheless, this perspective has left open to interpretation the dramatic similarity in sensory and internal connectivity between the avian DVR and the mammalian isocortex. One possibility is that in mammals, the collothalamic afferents were re-routed from the ventral pallium to the dorsal pallium, or that the embryonic territory originally destined to the DVR (lateral or ventral pallium) became phenotypically transformed into dorsal pallium, however maintaining its collothalamic sensory inputs (see below; Aboitiz et al., 2002, 2003b). Another interpretation is that the collothalamic projections to the DVR and the isocortex are not really homologous, there being a collothalamic projection to the mammalian amygdala which would be the most likely homolog of the input to the sauropsidian DVR (Puelles, 2001).

## Patterning centers in pallial development and evolution

We recently developed an hypothesis that attempts to find a common ground for these dissenting interpretations, which prescribes the parallel amplification of a common, ancestral developmental program in the pallium of mammals and sauropsids, yielding brain expansion in both groups but differing in the embryological locus for the expansion (predominantly dorsal pallium in mammals; predominantly lateral/ventral pallium in sauropsids; Aboitiz, 2011). In order to make this hypothesis clearer, we will first discuss some evidence on the embryological development of the mammalian pallium, which is the taxon that has better been studied, particularly the mouse. Evidence to date suggests that these patterning processes are conservative across vertebrates, while we propose that subtle modulations in the absolute and relative activities of these different centers may yield dramatic changes in brain morphology while maintaining a conserved topographic organization.

### Patterning centers of the mammalian brain

**Figure 1:**
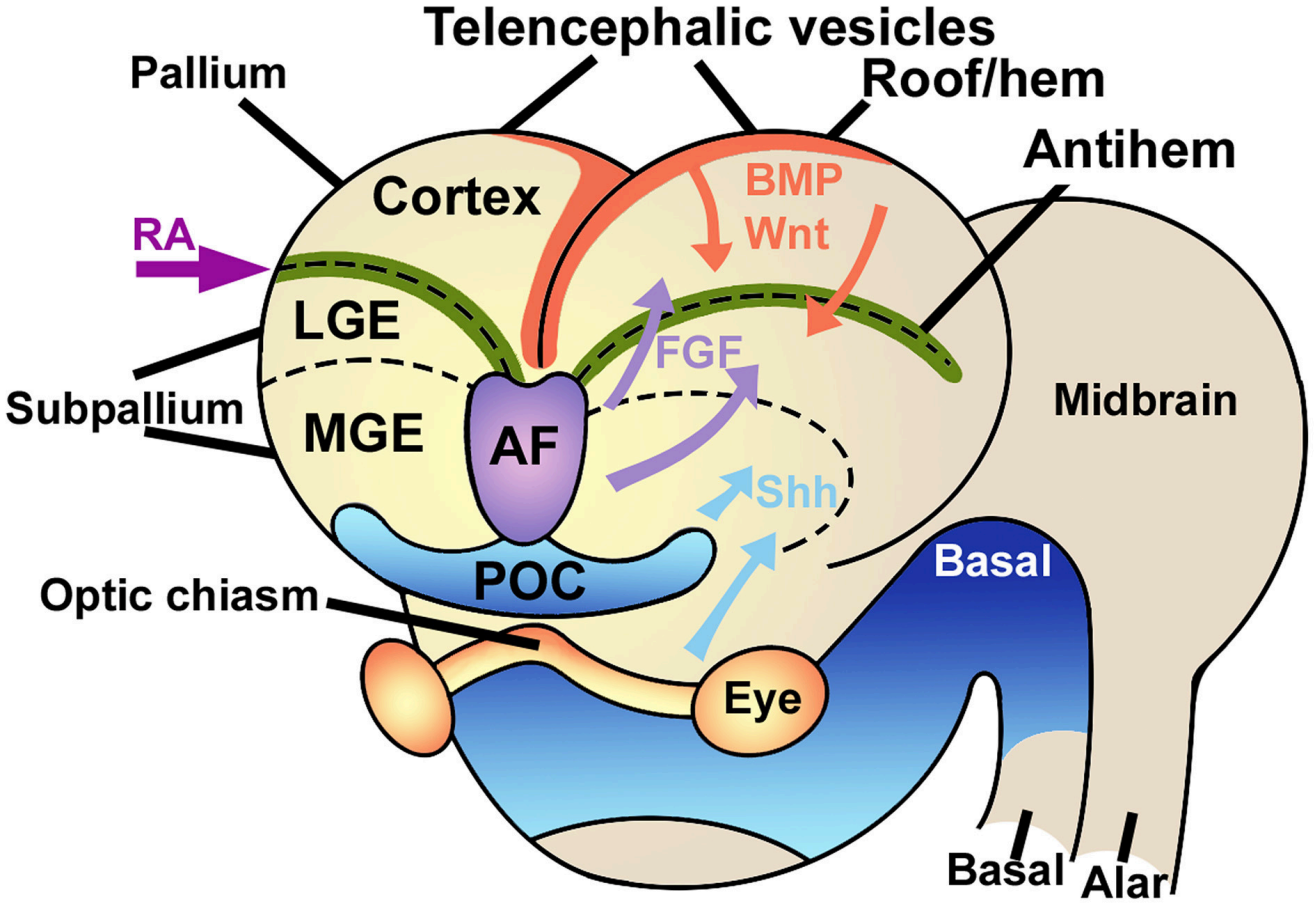
**Signaling centers in the embryonic brain**. The telencephalic vesicles or cerebral hemispheres are patterned by the combined action of different signaling centers like the rostral-patterning center in the anterior forebrain (AF, violet) secreting FGFs, the dorsal hem (red), secreting Wnts and BMPs, and the antihem (green), which specifies the ventral pallium. Other signaling elements are retinoic acid (RA) laterally and sonic hedgehog (Shh) ventrally. LGE, lateral ganglionic eminence; MGE, medial ganglionic eminence; POC, commissural preoptic area. Modified from Medina and Abellán (2009), Sur and Rubenstein (2005) and Aboitiz and Montiel (2012) with permission.

The embryonic pallium is patterned into distinct embryonic regions by at least three signaling centers, the cortical hem in the mediodorsal region, the antihem in the lateral aspect, and the anterior forebrain in the anteromedial region (Figure 1). All these centers secrete specific patterning molecules that diffuse in complementary gradients, generating a regional differentiation matrix that can be experimentally modulated by upregulation or downregulation of each of these signaling centers, yielding expansions of some pallial regions at the expense of others, or transforming the phenotypic identity of some areas (Shimogori et al., 2004).

**Figure 2:**
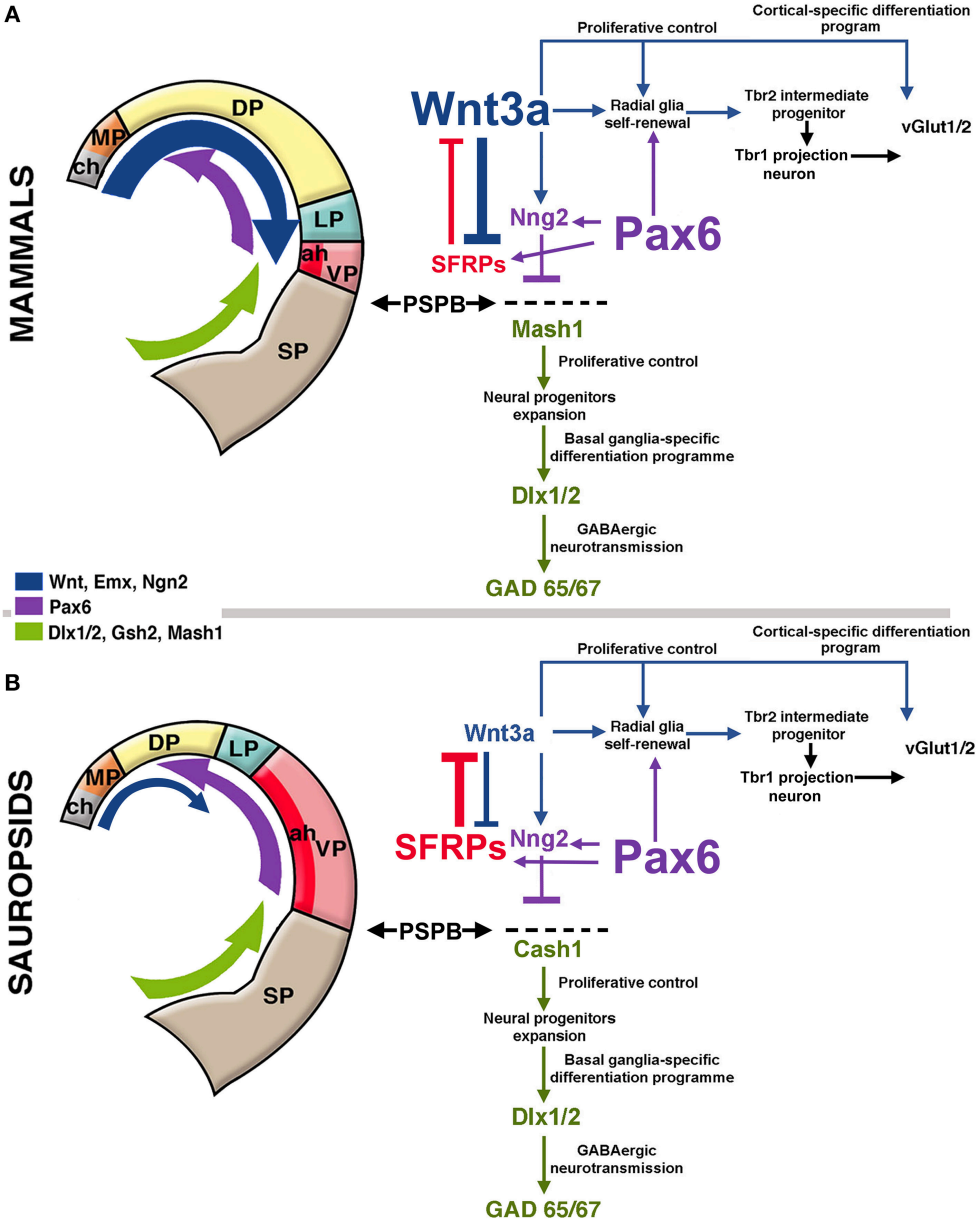
**Hypothetical model of telencephalic signaling to drive pallial expansion in mammals and sauropsids**. **(A)** In mammals, dorsal signaling upregulation (blue arrow in the figure) expands the dorsal pallium (DP). In early stages, the upregulation of Wnt3a from the cortical hem (ch, gray in figure) activates the self-renewal of radial glia. In addition, upregulation of ventral factors like Pax6 (purple arrow in the figure) induce late proliferation of radial glia that generate Tbr2+ intermediate progenitors in the SVZ (see diagram at the right). Pax6 also contributes to the formation of the antihem (ah, dark red) that expresses secreted Frizzled-Related Proteins (Sfrp1 and 2; in red) that neutralize the action of dorsally-derived signals like Wnts. Pax6 also activates the expression of the proneural factor neurogenin 1/2 (Sansom et al., 2009) with the consequent inhibition of Mash1 (Cash1 in chicken), a proneural factor highly expressed in subpallial domains. Mash1 induces a cascade that leads to subpallial neural phenotypes (Castro et al., 2011). The pallial/subpallial boundary (PSPB) is defined by the limit of expression of Ngn2 and Mash1 genes (black arrows). **(B)** In sauropsids, the dorsalizing activity of the cortical hem remains reduced, but like in mammals, there is an upregulation of Pax6, leading to the expansion of the antihem as there is little dorsal activity to counteract it. While in mammals there is a strong superposition of lateral/ventral signals and dorsal signals, in sauropsids lateral/ventral signals tend to be more decisive for pallial patterning.

Specifically, the cortical hem secretes morphogens like BMPs and Wnts, that determine the development of the medial pallium (hippocampus) and establish a posteromedial/high to anterolateral/low gradient along the dorsal pallium, regulating proliferation and patterning of these two structures (Shimogori et al., 2004; O'Leary et al., 2007; Harrison-Uy and Pleasure, 2012). Dorsally derived Wnt factors induce progenitor proliferation at early stages, but are later replaced by the ventrally derived morphogen Pax6 to maintain progenitor cell division (Zhou et al., 2006; Machon et al., 2007; Kuwahara et al., 2010; Harrison-Uy and Pleasure, 2012; Figure 2).

Caronia-Brown et al. (2014) recently reported that the cortical hem is a key regulator of both the size and patterning of the neocortex beside the hippocampus, where mice with cortical hem ablations displayed a reduced caudal and dorsomedial neocortex, while the rostral and ventrolateral neocortices expanded normally or increased in relative size. Wnt3a signal from the cortical hem induces self-renewal of radial glia and the early differentiation of cortical intermediate progenitors via the canonical Wnt pathway (Munji et al., 2011). Both opposing roles are modulated by Hipk1 in a dose-dependent mechanism (Marinaro et al., 2012). In the mouse, upregulation of Wnt3a activates the production of intermediate progenitors from radial glial cells increasing the Tbr2-positive cell populations (Kuwahara et al., 2010; Munji et al., 2011; Figure 2A). In the Tbr2 KO mouse, the interruption of this cascade leads to a reduction of the intermediate progenitors and a cortical size reduction (Arnold et al., 2008). It has also been found that the transcription factor Lhx2 constraints the development of both the cortical hem and Cajal-Retzius cells, at the expense of favoring the development of the cortical neuroepithelium including the hippocampus (Bulchand et al., 2001; Roy et al., 2014).

Of note, the corpus callosum, the largest tract in the brain and a unique character of placental mammals, originates dorsally to the hippocampal commissure common to all vertebrates (Aboitiz and Montiel, 2003; Aboitiz et al., 2003a). Marsupials and monotremes also have interhemispheric connections (which are actually unique to mammals), but these cross through the anterior commissure located in the anterior forebrain. Recently, the cellular and molecular mechanisms involved in the generation of the corpus callosum have been analyzed by the group of Linda Richards, implying a critical role of the commissural plate, particularly the cortical hem, in establishing the anatomical and molecular substrate for the development of interhemispheric connections along the nascent corpus callosum (Suárez et al., 2014).

More laterally in the pallium, the antihem expresses different EGFs, FGFs and especially Frizzled-related proteins that antagonize the effects of dorsal morphogens secreted by the cortical hem, and are considered to specify the ventral pallial territory (Assimacopoulos et al., 2003; Figure 2). Noticeably, Pax6 signaling induces the antihem, and is strongly expressed in the ventral pallium and equatorial aspect of the hemisphere, showing a gradient of activity from the anterolateral to the caudomedial hemisphere. Pax6 is required for the development of lateral and ventral pallial identities (olfactory and amygdalar components; Piñon et al., 2008; Cocas et al., 2011), and patterns the anterolateral neocortex (Bishop et al., 2000). Pax6 is a critical promotor of progenitor proliferation, and an essential component for the evolutionary expansion of the neocortex (Poluch and Juliano, 2015). The developmental expression pattern of signaling factors is mostly conserved between chick and mouse, but a broader expression of Pax6 is detected in the ventral pallium of chick (Frowein et al., 2002; Assimacopoulos et al., 2003). For this reason, we proposed this to be a prime candidate for a proliferative signal in all amniotes (Aboitiz, 2011; Aboitiz and Zamorano, 2013), triggering a conserved differentiation cascade in both sauropsids and mammals. Additional candidates to stimulate progenitor amplification are regulators of Notch, which controls the stem cell cycle and neurogenesis and is highly expressed in reptiles like the gecko (Nomura et al., 2013, 2014), POU homeobox factors (Dominguez et al., 2013) and other proteins that regulate progenitor proliferation (Vied et al., 2014).

The third patterning center, located in the anterior forebrain is the rostral patterning center (RPC), which secretes molecules of the FGF family and promotes ventral and anterior telencephalic fates (Rubenstein, 2011). FGF signaling from the RPC has a strong role in patterning not only the neocortex but also the diencephalon and ventral telencephalon, In addition, FGF signaling is required for the generation of commissural connectivity as well as differentiation of the dorsal midline (Shanmugalingam et al., 2000).

From a comparative perspective, the RPC may be closely related to the anterior neural ridge, located in the frontal edge of the head, in front of the most anterior neural tissue, which is also characterized by strong expression of FGF8 and other members of the FGF family (Pownall and Isaacs, 2010). The anterior neural ridge induces the olfactory and adenohypophyseal placodes, both of which apparently derive from a primitive panplacodal primordium (Schlosser, 2005). The role of FGF signaling in telencephalic patterning has been observed even in basal vertebrates like the lamprey (Sugahara et al., 2011), which indicates its conservatism in evolution, possibly in association with the evolution of olfaction (Aboitiz and Montiel, 2007a,b).

The RPC expresses a variety of FGF factors, of which FGF8 has been the most studied in relation to forebrain and cortical patterning. During embryogenesis, FGF8 diffuses caudally from the anterior forebrain, promoting ventral and anterior phenotypes and inducing progenitor proliferation (Borello et al., 2008; Toyoda et al., 2010). FGF8 induces the expression of FOXG1, which antagonizes the activity of the dorsal morphogen BMP (Shimamura and Rubenstein, 1997). Furthermore, FGF8 is strongly required for frontal cortex differentiation, and while hypomorphic expression in mutant mice leads to reductions in frontal cortex size and expansion of caudal markers, its ectopic overexpression has resulted in the generation of an additional somatosensory cortex and thalamus (Fukuchi-Shimogori and Grove, 2001). Noteworthy, FGF8 can regulate postnatal thalamic innervation and the intracortical wiring pattern, even if the initial connectivity pattern is not affected in newborn FGF8 mutants (see Danjo et al., 2011). Other members of the FGF family have been found to have similar effects in brain development. For example, like FGF8, mutations in FGF17 produce frontal, midbrain and cerebellar alterations, as well as behavioral deficits reminiscent of autistic spectrum symptomatology. Furthermore, progenitors generated in the RPC contribute neurons to wide regions of the telencephalon, including medial prefrontal cortex (Hoch et al., 2015). There is evidence that FGF2-sensitive neural stem cells (expressing Fgfr1) are required for hippocampal growth (Ohkubo et al., 2004), but it is not clear that FGF2 derives from the RPC (Rubenstein, 2011).

Finally, the cortical hem, the antihem and the anterior forebrain (but particularly the cortical hem), are the sites of generation of Cajal-Retzius cells that secrete the glycoprotein reelin, required for proper neocortical lamination and dendritic growth of pyramidal neurons (Nomura et al., 2008; Meyer, 2010; Kupferman et al., 2014; Martínez-Cerdeño and Noctor, 2014).

## A unifying hypothesis

Considering the above evidence, we have outlined an hypothesis that considers both the developmental and the phenotypic comparative evidence, thus attempting to account for both perspectives of pallial homology in amniotes (Aboitiz, 2011; Aboitiz and Montiel, 2012; Aboitiz and Zamorano, 2013). This hypothesis is based on the differential modulation of telencephalic patterning centers in sauropsids and mammals, and, as we suggest in this article, on the overlap of distinct morphogenetic fields only in mammals, which yielded the expansion of the isocortex in this group.

### Shared, Pax6- dependent brain amplification

We have proposed that the expansion of both the avian pallium and the mammalian isocortex relied on cascades driven by several, phylogenetically conserved neurogenetic factors. One likely candidate is Pax6, which promotes progenitor division and the extension of neurogenesis. Pax6 is maximally expressed in lateral and ventral pallial regions, decreasing its expression in the rostrocaudal direction. In addition to this spatial gradient, there is also a temporal gradient of Pax6 expression from the anteroventral to the rostrocaudal pallium (Aboitiz et al., 2003b; Aboitiz and Montiel, 2007b, 2012; Aboitiz and Zamorano, 2013). The conservation of this signaling cascade may explain the phenotypic concordance of lateral and ventral pallial cells in the sauropsidian brain and cells in the upper isocortical layers (derived from the dorsal pallium). The latter are considered to be, in general, phylogenetically newer than lower isocortical layers as they derive from the embryonic subventricular zone (SVZ), a compartment for late progenitor proliferation that is found in the embryonic precursors of both the avian DVR and the mammalian isocortex (Reiner, 1991; Cheung et al., 2007; for reviews see Aboitiz et al., 2001, 2003b). The SVZ develops due to an amplification of Pax6 signaling in the radial glia of the VZ, whose progeny migrates to the SVZ and expresses the marker Tbr2 (see Englund et al., 2005; Ypsilanti and Rubenstein, 2015). Recently, Martínez-Cerdeño et al. (2015) have shown that in the turtle and avian DVR (lateral/ventral pallium), and in the mammalian isocortex, Pax6-expressing radial glia give rise to Tbr2-expressing intermediate progenitors that migrate into the SVZ. Most notably, in the DVR of the lizard and in the dorsal cortex of turtle and lizard, no distinct SVZ could be seen, while scattered Tbr2+ cells could be found in the VZ (Montiel et al., 2015). This resembles early developmental stages in mammals, where Tbr2+ cells can be found in the VZ before the SVZ becomes a distinct layer (Noctor et al., 2008). This evidence is consistent with our previous hypothesis of a conserved program of brain expansion in amniotes (Aboitiz, 2011; Aboitiz and Montiel, 2012; Aboitiz and Zamorano, 2013).

### Overlap between dorsal and ventral morphogenetic fields in mammals

Thus, there is evidence supporting a conserved developmental schedule in the pallium of amniotes (and possibly in other vertebrates). But this leaves open a fundamental question that has not yet been properly addressed: why in mammals a laminated structure became established, while in sauropsids the non-laminar condition prevails? In our view, this results from the additional amplification of the dorsal and anterior signaling centers (the cortical hem and RPC), that together with the proliferation of Cajal-Retzius cells, promoted a pyramidal morphology in excitatory cells, and a columnar organization to the isocortex. Thus, in addition to the latero-ventral pallial driven amplification of progenitor proliferation, the mammalian brain would have suffered a process of “dorsalization” in its development, where dorsal signaling factors became upregulated and determined a conspicuous laminar organization in the dorsal pallium, aided by the amplification of reelin-producing Cajal-Retzius cells (Aboitiz et al., 2003b). In this account, it is conceivable that the boundaries between the dorsal and the lateral/ventral pallium became shifted so that territory originally destined to the future DVR became partially transformed into a cortical phenotype, while maintaining its original collothalamic afferences. As said, another option is that collothalamic afferents were re-routed into the expanding dorsal pallium (Aboitiz et al., 2002, 2003b). Overall, this hypothesis has the virtue of reconciling the developmental and the phenotypic evidences into one overarching developmental-evolutionary process.

Overall, the point is that a similar developmental cascade to enhance progenitor proliferation and increasing neuronal numbers (presumably depending on Pax6 and associated with the development of a SVZ) became activated in both birds and mammals. This process took place in the lateral and ventral pallium of birds, and in the dorsal pallium of mammals, in the latter contributing to the generation of late-produced superficial isocortical layers. Our hypothesis implies that although Pax6 has been upregulated in both lineages, only in mammals there is a concomitant upregulation of the cortical hem that limits the expansion of the antihem but has no strong effect in Pax6 activity (Aboitiz, 2011; Aboitiz and Montiel, 2012; Aboitiz and Zamorano, 2013). In fact, there is a superposition of dorsal, hem-related signals like Emx2 and Pax6 in the developing neocortex, which contribute to aeral patterning of this structure (O'Leary and Sahara, 2008).

### Temporal segregation between dorsal and ventral patterning centers

Some authors have advanced the concept of a spatial-to-temporal transformation of the differentiation programs of neuronal types in sauropsids and mammals (Nomura et al., 2009; Suzuki et al., 2012). Thus, neurons in the more conservative mediodorsal pallium of sauropsids tend to express markers that are also found in lower isocortical layers in mammals; while mammalian mid- and upper isocortical markers tend to be found in the most expanding ventral, lateral and dorsal pallial regions (for more details see Table 1 in Aboitiz and Zamorano, 2013); see also (Nomura et al., 2009; Suzuki et al., 2012; Belgard et al., 2013). An additional hypothesis has been put forward by Federico Luzzati (2015), who noticed a similar expression of markers like DCX/Tbr1 in the lateral (olfactory) cortex of reptiles and the superficial layers of the mammalian isocortex. Luzzati proposes that the emerging, dorsal pallial, mammalian isocortex co-opted a lateral pallial developmental program to generate the superficial isocortical layers, a possibility that is in general terms consistent with ours.

Thus, deep isocortical layers show a different phenotype than the superficial ones, which share more markers with the lateral pallial neurons of sauropsids. In this line, there may be a differential timing in the activity of hem-derived transcription factors and of Pax6, especially considering that the cortical hem has been found to be a strong regulator of the size of the neocortex (Caronia-Brown et al., 2014) and that Wnts induce progenitor proliferation at early stages (Zhou et al., 2006; Machon et al., 2007; Kuwahara et al., 2010; Harrison-Uy and Pleasure, 2012). Early Wnt activity (or other dorsal factors) might contribute to specify the deep layer isocortical neurons, with dorsal pallial phenotypes. On the other hand, there are different Pax6 transcripts expressed in different developmental stages, with partly antagonist activities among them, that may fine-regulate the extent of progenitor proliferation (Ypsilanti and Rubenstein, 2015). Furthermore, a recent article reports the existence of a lineage-restricted population of radial glia that engages in neurogenic divisions only in late development, giving rise to neocortical supragranular neurons and particularly callosal-projecting neurons (García-Moreno and Molnár, 2015). Moreover, this type of late-engaging progenitors was not observed in sauropsids. It may be that early dorsalizing factors are producing a delay in the neurogenic activity of Pax6 activity, which becomes expressed in late development. The progeny of these late, Pax6-driven cells, might share features with early produced neurons in the lateral pallium, as proposed by several authors (Nomura et al., 2009; Suzuki et al., 2012; Luzzati, 2015). Finally, the hem-derived Cajal-Retzius cells might also contribute to laminar specification in late developmental stages, promoting a columnar organization of the cells of the cortical plate.

### Genetic but not regional homology

The main discussion regarding the comparisons of avian and mammalian brains relies on the issue of homology, i.e., to what extent these similarities can be tracked to a common ancestor. In our opinion, homology is not an all-or-none condition but depends on the phenomenical level at which it is observed (De Beer, 1971; Aboitiz, 1988; Striedter and Northcutt, 1991). There may not be regional homology between the isocortex and the DVR as both structures derive from different embryonic regions (dorsal pallium and lateral/ventral pallium, respectively). Nonetheless, some early embryonic territory destined to the lateral-ventral pallium may have acquired a dorsal pallial identity by influence of the expanding cortical hem, which again would cast doubts about the strict meaning of homology (i.e., do the progenitor cell populations or the developmental fields determine homology?).

On the other hand, the genetic cascades involved in brain growth are partly the same, regardless of pallial region (lateroventral pallium in sauropsids, dorsal pallium in mammals); and they presumably depend on Pax6 signaling to amplify the progenitor cell population. In other words, an upregulation of Pax6 or related signals was independently recruited in birds and mammals to amplify progenitor cell population, an instance of co-option of a shared developmental program in a new context (Aboitiz and Montiel, 2007a). Thus, in the common ancestor, the morphogenetic fields specified by Pax6 activity (including the antihem), the cortical hem and the anterior forebrain may have suffered little overlap, generating a spatial, or tangential gradient of neuronal differentiation. This situation was probably maintained in reptiles and to some extent also in birds. Nonetheless, in mammals this becomes complicated by the additional influence of the cortical hem and anterior forebrain. Concurrent amplification of all these signaling centers in early mammals yielded an extensive overlap between their respective morphogenetic fields, and contributed to the establishment of a temporal, or radial gradient of differentiation in the nascent isocortex. Dorsalizing factors acted at early stages, determining the phenotypes of early-produced inferior layer neurons, while Pax6 amplification exerted its effects at later stages, which together with Cajal-Retzius cells determined the phenotypes of late-produced, superficial layer neurons. There is some evidence that is consistent with this view, as the cortical hem is present but is much less developed in the sauropsids that have been studied than in mammals, showing decreased specific markers like cWnt8b and a much smaller population of Cajal-Retzius cells (Cabrera-Socorro et al., 2007; Medina and Abellán, 2009; Subramanian et al., 2009; Abellán et al., 2010). In birds and reptiles, there are scattered cells with a Cajal-Retzius typical morphology, but these are not nearly as abundant as in mammals and express much lower levels of reelin. Finally, this proposal predicts that as the isocortex increases in size by amplification of genes like Pax6 and others, there is a growing influence from the cortical hem to maintain its laminar structure and patterning (Tarabykin et al., 2001; Tuoc et al., 2009; Caronia-Brown et al., 2014).

### Hippocampus and olfactory cortex

The cortical hem is also critical for the development of the mammalian dentate gyrus and hippocampus, components that have evidenced a significant increase in size and complexity in mammals (Hevner, 2015), although not comparable with the expansion of the isocortex. Thus, there is still the question of why did the former structures, that depend most directly on the cortical hem, did not expand explosively as the isocortex did. It is possible that there are some yet unknown factors, perhaps related to a decreased activity of Pax6 signaling in dorsomedial regions, or to activity from the RPC, that may restrict the expansion of the embryonic medial pallium (hippocampus and dental gyrus), but at the same time be permissive for dorsal pallial expansion (giving rise to the isocortex).

Likewise, early mammals displayed a moderate expansion of the olfactory cortex before the isocortex took off (Rowe and Shepherd, 2015). We suggest that the early expansion of both, the mammalian olfactory cortex and the reptilian DVR, was probably driven by a moderate upregulation of Pax6 activity in both groups. However, in early mammals and reptiles, there may have been different selective pressures on sensory processing: olfaction in mammals and vision in reptiles. Perhaps natural selection favored the activation of distinct Pax6-dependent, tissue-specific enhancers in mammals and reptiles (see Ypsilanti and Rubenstein, 2015), that promoted the development of the olfactory cortex in mammals and the DVR in reptiles, to support vision in the latter. In later stages of mammalian evolution, Wnt activity became upregulated, leading isocortical expansion, and restricting the relative sizes of the olfactory cortex and amygdala, as evidenced by their inverse scaling with isocortical size across mammals (Reep et al., 2007).

Although significant, the expansion of the olfactory cortex in early mammals was limited for at least two reasons: (1) The brain of mammal-like ancestors was already small, and (2) There are functional limits to the radial expansion of the olfactory cortex, which relies strongly in tangential, associative interactions (Rowe and Shepherd, 2015). Although the neocortex inherits the same tangential organization, it superimposes a radial arrangement over it, associated to the development of a SVZ (see Bosman and Aboitiz, 2015).

## Summary

In this article, we have reviewed developmental evidence supporting the concept that the origin of the mammalian brain relies on the amplification of several morphogenetic centers that participate in patterning the dorsal cerebral hemisphere or pallium. Furthermore, we claim that there are conserved molecular mechanisms for progenitor cell division and neuronal differentiation at least in all amniotes, which may rely on a cascade associated to Pax6 and other genes, which act in a lateral-to-dorsomedial gradient thereby tending to differentiate and augment ventral and lateral pallial phenotypes. However, mammals underwent a diverging trend by, in addition, enhancing the activity of dorsomedial and anterior telencephalic signaling centers (the anterior forebrain and the cortical hem, respectively) that, together with the proliferation of reelin-producing Cajal-Retzius cells, induced a laminar arrangement and a characteristic pyramidal cell shape for excitatory neurons in the medial and dorsal pallium (note that a rudimentary laminar arrangement of pyramidal cells already exists in the cortex of reptiles and in the olfactory cortex of mammals). Morphogens derived from these centers also restricted the expansion of the antihem in the lateral and ventral pallium, and favored the generation of a dorsal pallial neocortex that was initially small, with a relatively large olfactory cortex, as in basal therian mammals. Thus, a differentiation gradient that was ancestrally established in the tangential axis, became expressed in the radial domain by virtue of the superposition of the different signaling molecules that acted at different developmental stages, i.e., dorsal-derived Wnts at early stages, and laterally-derived Pax6 signals at late developmental stages. In subsequent lineages, the isocortex expanded enormously both in absolute and relative size. In line with these arguments, Lewitus et al. (2014) have recently proposed that the ancestor of crown mammals might have had a gyrencephalic brain with a well differentiated isocortex, which would imply that the origin of isocortex is to be traced back to earlier mammalian groups, possibly living in the Jurassic period (Luo, 2007; Lee and Beck, 2015).

## Conflict of interest statement

The authors declare that the research was conducted in the absence of any commercial or financial relationships that could be construed as a potential conflict of interest.

## References

[B1] Abellán A. Legaz I. Vernier B. Rétaux S. Medina L. (2009). Olfactory and amygdalar structures of the chicken ventral pallium based on the combinatorial expression patterns of LIM and other developmental regulatory genes. J. Comp. Neurol. 516, 166–186. 10.1002/cne.22102 19598282

[B2] Abellán A. Menuet A. Dehay C. Medina L. Rétaux S. (2010). Differential expression of LIM-homeodomain factors in Cajal-Retzius cells of primates, rodents, and birds. Cereb. Cortex 20, 1788–1798. 10.1093/cercor/bhp242 19923199

[B3] Aboitiz F. (1988). Homology: a comparative or a historical concept? Acta Biotheor. 37, 27–29. 3149101 10.1007/BF00050805

[B4] Aboitiz F. (1992). The evolutionary origin of the mammalian cerebral cortex. Biol. Res. 25, 41–49. 1341579

[B5] Aboitiz F. (2011). Genetic and developmental homology in amniote brains. Toward conciliating radical views of brain evolution. Brain Res. Bull. 84, 125–136. 10.1016/j.brainresbull.2010.12.003 21146594

[B6] Aboitiz F. López J. Montiel J. (2003a). Long distance communication in the human brain: timing constraints for inter-hemispheric synchrony and the origin of brain lateralization. Biol. Res. 36, 89–99. 10.4067/S0716-97602003000100007 12795208

[B7] Aboitiz F. Montiel J. (2003). One hundred million years of interhemispheric communication: the history of the corpus callosum. Braz. J. Med. Biol. Res. 36, 409–420. 10.1590/S0100-879X2003000400002 12700818

[B8] Aboitiz F. Montiel J. (2007a). Co-option of signaling mechanisms from neural induction to telencephalic patterning. Rev. Neurosci. 18, 311–342. 10.1515/REVNEURO.2007.18.3-4.311 18019612

[B9] Aboitiz F. Montiel J. (2007b). Origin and evolution of the vertebrate telencephalon, with special reference to the mammalian neocortex. Adv. Anat. Embryol. Cell Biol. 193, 1–112. 10.1007/978-3-540-49761-5 17595827

[B10] Aboitiz F. Montiel J. F. (2012). From tetrapods to primates: conserved developmental mechanisms in diverging ecological adaptations. Prog. Brain Res. 195, 3–24. 10.1016/B978-0-444-53860-4.00001-5 22230620

[B11] Aboitiz F. Montiel J. Morales D. Concha M. (2002). Evolutionary divergence of the reptilian and the mammalian brains: considerations on connectivity and development. Brain Res. Brain Res. Rev. 39, 141–153. 10.1016/S0165-0173(02)00180-7 12423764

[B12] Aboitiz F. Morales D. Montiel J. (2001). The inverted neurogenetic gradient of the mammalian isocortex: development and evolution. Brain Res. Brain Res. Rev. 38, 129–139. 10.1016/S0006-8993(01)02902-X 11750929

[B13] Aboitiz F. Morales D. Montiel J. (2003b). The evolutionary origin of the mammalian isocortex: towards an integrated developmental and functional approach. Behav. Brain Sci. 26, 535–552. discussion 552–585. 10.1017/S0140525X0326012X 15179935

[B14] Aboitiz F. Zamorano F. (2013). Neural progenitors, patterning and ecology in neocortical origins. Front. Neuroanat. 7:38. 10.3389/fnana.2013.00038 24273496 PMC3824149

[B15] Ahumada-Galleguillos P. Fernández M. Marin G. J. Letelier J. C. Mpodozis J. (2015). Anatomical organization of the visual dorsal ventricular ridge in the chick (*Gallus gallus*): layers and columns in the avian pallium. J. Comp. Neurol. 523, 2618–2636. 10.1002/cne.23808 25982840

[B16] Ariëns Kappers C. V. Huber C. G. Crosby E. C. (1936). The Comparative Anatomy of the Nervous System of Vertebrates, Including Man. New York, NY: Hafner.

[B17] Arnold S. J. Huang G. J. Cheung A. F. P. Era T. Nishikawa S. I. Bikoff E. K.. (2008). The T-box transcription factor Eomes/Tbr2 regulates neurogenesis in the cortical subventricular zone. Genes Dev. 22, 2479–2484. 10.1101/gad.475408 18794345 PMC2546697

[B18] Assimacopoulos S. Grove E. A. Ragsdale C. W. (2003). Identification of a Pax6-dependent epidermal growth factor family signaling source at the lateral edge of the embryonic cerebral cortex. J. Neurosci. 23, 6399–6403. 10.1002/cne.903070312 12878679 PMC6740631

[B19] Belgard T. G. Montiel J. F. Wang W. Z. García-Moreno F. Margulies E. H. Ponting C. P.. (2013). Adult pallium transcriptomes surprise in not reflecting predicted homologies across diverse chicken and mouse pallial sectors. Proc. Natl. Acad. Sci. U.S.A. 110, 13150–13155. 10.1073/pnas.1307444110 23878249 PMC3740902

[B20] Bishop K. M. Goudreau G. O'Leary D. D. (2000). Regulation of area identity in the mammalian neocortex by Emx2 and Pax6. Science 288, 344–349. 10.1126/science.288.5464.344 10764649

[B21] Borello U. Cobos I. Long J. E. McWhirter J. R. Murre C. Rubenstein J. L. R. (2008). FGF15 promotes neurogenesis and opposes FGF8 function during neocortical development. Neural Dev. 3:17. 10.1186/1749-8104-3-17 18625063 PMC2492847

[B22] Bosman C. A. Aboitiz F. (2015). Functional constraints in the evolution of brain circuits. Front. Neurosci. 9:303. 10.3389/fnins.2015.00303 26388716 PMC4555059

[B23] Bruce L. L. Neary T. J. (1995). The limbic system of tetrapods: a comparative analysis of cortical and amygdalar populations. Brain Behav. Evol. 46, 224–234. 10.1159/000113276 8564465

[B24] Bulchand S. Grove E. A. Porter F. D. Tole S. (2001). LIM-homeodomain gene Lhx2 regulates the formation of the cortical hem. Mech. Dev. 100, 165–175. 10.1016/S0925-4773(00)00515-3 11165475

[B25] Butler A. B. (1995). The dorsal thalamus of jawed vertebrates - a comparative viewpoint. Brain Behav. Evol. 46, 209–223. 10.1159/000113275 8564464

[B26] Butler A. B. Reiner A. Karten H. J. (2011). Evolution of the amniote pallium and the origins of mammalian neocortex. Ann. N. Y. Acad. Sci. 1225, 14–27. 10.1111/j.1749-6632.2011.06006.x 21534989 PMC3384708

[B27] Cabrera-Socorro A. Hernandez-Acosta N. C. Gonzalez-Gomez M. Meyer G. (2007). Comparative aspects of p73 and Reelin expression in Cajal-Retzius cells and the cortical hem in lizard, mouse and human. Brain Res. 1132, 59–70. 10.1016/j.brainres.2006.11.015 17189620

[B28] Calabrese A. Woolley S. M. N. (2015). Coding principles of the canonical cortical microcircuit in the avian brain. Proc. Natl. Acad. Sci. U.S.A. 112, 3517–3522. 10.1073/pnas.1408545112 25691736 PMC4371993

[B29] Caronia-Brown G. Yoshida M. Gulden F. Assimacopoulos S. Grove E. A. (2014). The cortical hem regulates the size and patterning of neocortex. Development 141, 2855–2865. 10.1242/dev.106914 24948604 PMC4197624

[B30] Castro D. S. Martynoga B. Parras C. Ramesh V. Pacary E. Johnston C.. (2011). A novel function of the proneural factor Ascl1 in progenitor proliferation identified by genome-wide characterization of its targets. Genes Dev. 25, 930–945. 10.1101/gad.627811 21536733 PMC3084027

[B31] Cheung A. F. P. Pollen A. A. Tavare A. DeProto J. Molnár Z. (2007). Comparative aspects of cortical neurogenesis in vertebrates. J. Anat. 211, 164–176. 10.1111/j.1469-7580.2007.00769.x 17634059 PMC2375772

[B32] Cocas L. A. Georgala P. A. Mangin J.-M. Clegg J. M. Kessaris N. Haydar T. F.. (2011). Pax6 is required at the telencephalic pallial-subpallial boundary for the generation of neuronal diversity in the postnatal limbic system. J. Neurosci. 31, 5313–5324. 10.1523/JNEUROSCI.3867-10.2011 21471366 PMC3086773

[B33] Danjo T. Eiraku M. Muguruma K. Watanabe K. Kawada M. Yanagawa Y.. (2011). Subregional specification of embryonic stem cell-derived ventral telencephalic tissues by timed and combinatory treatment with extrinsic signals. J. Neurosci. 31, 1919–1933. 10.1523/JNEUROSCI.5128-10.2011 21289201 PMC6623725

[B34] De Beer G. (1971). Homology, an Unsolved Problem. Glasgow: Oxford University Press.

[B35] Dominguez M. H. Ayoub A. E. Rakic P. (2013). POU-III transcription factors (Brn1, Brn2, and Oct6) influence neurogenesis, molecular identity, and migratory destination of upper-layer cells of the cerebral cortex. Cereb. Cortex 23, 2632–2643. 10.1093/cercor/bhs252 22892427 PMC3792741

[B36] Englund C. Fink A. Lau C. Pham D. Daza R. A. Bulfone A.. (2005). Pax6, Tbr2, and Tbr1 are expressed sequentially by radial glia, intermediate progenitor cells, and postmitotic neurons in developing neocortex. J. Neurosci. 25, 247–251. 10.1523/JNEUROSCI.2899-04.2005 15634788 PMC6725189

[B37] Frowein J. Campbell K. Götz M. (2002). Expression of Ngn1, Ngn2, Cash1, Gsh2 and Sfrp1 in the developing chick telencephalon. Mech. Dev. 110, 249–252. 10.1016/S0925-4773(01)00590-1 11744393

[B38] Fukuchi-Shimogori T. Grove E. A. (2001). Neocortex patterning by the secreted signaling molecule FGF8. Science 294, 1071–1074. 10.1126/science.1064252 11567107

[B39] Gao P. Postiglione M. P. Krieger T. G. Hernandez L. Wang C. Han Z.. (2014). Deterministic progenitor behavior and unitary production of neurons in the neocortex. Cell 159, 775–788. 10.1016/j.cell.2014.10.027 25417155 PMC4225456

[B40] García-Moreno F. Molnár Z. (2015). Subset of early radial glial progenitors that contribute to the development of callosal neurons is absent from avian brain. Proc. Natl. Acad. Sci. U.S.A. 112, E5058–E5067. 10.1073/pnas.1506377112 26305942 PMC4568669

[B41] Harrison-Uy S. J. Pleasure S. J. (2012). Wnt signaling and forebrain development. Cold Spring Harb. Perspect. Biol. 4:a008094. 10.1101/cshperspect.a008094 22621768 PMC3385962

[B42] Hevner R. F. (2015). Evolution of the mammalian dentate gyrus. J. Comp. Neurol. [Epub ahead of print]. 10.1002/cne.23851 26179319 PMC4706817

[B43] Hoch R. V. Clarke J. A. Rubenstein J. L. (2015). Fgf signaling controls the telencephalic distribution of Fgf-expressing progenitors generated in the rostral patterning center. Neural Dev. 10, 8. 10.1186/s13064-015-0037-7 25889070 PMC4416298

[B44] Holmgren N. (1922). Points of view concerning forebrain morphology in lower vertebrates. J. Comp. Neurol. 34, 391–459. 10.1002/cne.900340502

[B45] Jarvis E. D. Güntürkün O. Bruce L. Csillag A. Karten H. Kuenzel W.. (2005). Avian brains and a new understanding of vertebrate brain evolution. Nat. Rev. Neurosci. 6, 151–159. 10.1038/nrn1606 15685220 PMC2507884

[B46] Jarvis E. D. Yu J. Rivas M. V. Horita H. Feenders G. Whitney O.. (2013). Global view of the functional molecular organization of the avian cerebrum: mirror images and functional columns. J. Comp. Neurol. 521, 3614–3665. 10.1002/cne.23404 23818122 PMC4145244

[B47] Karten H. J. (1969). The organization of the avian telencephalon and some speculations on the phylogeny of the amniote telencephalon. Ann. N.Y. Acad. Sci. 167, 164–179. 10.1111/j.1749-6632.1969.tb20442.x

[B48] Karten H. J. (2013). Neocortical evolution: neuronal circuits arise independently of lamination. Curr. Biol. 23, R12–R15. 10.1016/j.cub.2012.11.013 23305661

[B49] Kupferman J. V. Basu J. Russo M. J. Guevarra J. Cheung S. K. Siegelbaum S. A. (2014). Reelin signaling specifies the molecular identity of the pyramidal neuron distal dendritic compartment. Cell 158, 1335–1347. 10.1016/j.cell.2014.07.035 25201528 PMC4183142

[B50] Kuwahara A. Hirabayashi Y. Knoepfler P. S. Taketo M. M. Sakai J. Kodama T.. (2010). Wnt signaling and its downstream target N-myc regulate basal progenitors in the developing neocortex. Development 137, 1035–1044. 10.1242/dev.046417 20215343

[B51] Lee M. S. Y. Beck R. M. D. (2015). Mammalian evolution: a Jurassic spark. Curr. Biol. 25, pR759–pR761. 10.1016/j.cub.2015.07.008 26325137

[B52] Lewitus E. Kelava I. Kalinka A. T. Tomancak P. Huttner W. B. (2014). An adaptive threshold in mammalian neocortical evolution. PLoS Biol. 12:e1002000. 10.1371/journal.pbio.1002000 25405475 PMC4236020

[B53] Luo Z.-X. (2007). Transformation and diversification in early mammal evolution. Nature 450, 1011–1019. 10.1038/nature06277 18075580

[B54] Luzzati F. (2015). A hypothesis for the evolution of the upper layers of the neocortex through co-option of the olfactory cortex developmental program. Front. Neurosci. 9:162. 10.3389/fnins.2015.00162 26029038 PMC4429232

[B55] Machon O. Backman M. Machonova O. Kozmik Z. Vacik T. Andersen L.. (2007). A dynamic gradient of Wnt signaling controls initiation of neurogenesis in the mammalian cortex and cellular specification in the hippocampus. Dev. Biol. 311, 223–237. 10.1016/j.ydbio.2007.08.038 17916349

[B56] Marinaro C. Pannese M. Weinandy F. Sessa A. Bergamaschi A. Taketo M. M.. (2012). Wnt signaling has opposing roles in the developing and the adult brain that are modulated by Hipk1. Cereb. Cortex 22, 2415–2427. 10.1093/cercor/bhr320 22095214

[B57] Martínez-Cerdeño V. Cunningham C. L. Camacho J. Keiter J. A. Ariza J. Lovern M.. (2015). Evolutionary origin of Tbr2-expressing precursor cells and the subventricular zone in the developing cortex. J. Comp. Neurol. [Epub ahead of print]. 10.1002/cne.23879 26267763 PMC4843790

[B58] Martínez-Cerdeño V. Noctor S. C. (2014). Cajal, Retzius, and Cajal-Retzius cells. Front. Neuroanat. 8:48. 10.3389/fnana.2014.00048 24987337 PMC4060955

[B59] Medina L. Abellán A. (2009). Development and evolution of the pallium. Semin. Cell Dev. Biol. 20, 698–711. 10.1016/j.semcdb.2009.04.008 19393324

[B60] Meyer G. (2010). Building a human cortex: the evolutionary differentiation of Cajal-Retzius cells and the cortical hem. J. Anat. 217, 334–343. 10.1111/j.1469-7580.2010.01266.x 20626498 PMC2992412

[B61] Montiel J. F. Molnár Z. (2013). The impact of gene expression analysis on evolving views on avian brain organization. J. Comp. Neurol. 521, 3604–3613. 10.1002/cne.23403 23818089

[B62] Montiel J. F. Vasistha N. A. Garcia-Moreno F. Molnár Z. (2015). From sauropsids to mammals and back: new approaches to comparative cortical development. J. Comp. Neurol. [Epub ahead of print]. 10.1002/cne.23871 26234252 PMC4832283

[B63] Munji R. N. Choe Y. Li G. Siegenthaler J. A. Pleasure S. J. (2011). Wnt signaling regulates neuronal differentiation of cortical intermediate progenitors. J. Neurosci. 31, 1676–1687. 10.1523/JNEUROSCI.5404-10.2011 21289176 PMC3040956

[B64] Noctor S. C. Martínez-Cerdeño V. Kriegstein A. R. (2008). Distinct behaviors of neural stem and progenitor cells underlie cortical neurogenesis. J. Comp. Neurol. 508, 28–44. 10.1002/cne.21669 18288691 PMC2635107

[B65] Nomura T. Gotoh H. Ono K. (2013). Changes in the regulation of cortical neurogenesis contribute to encephalization during amniote brain evolution. Nat. Commun. 4, 2206. 10.1038/ncomms3206 23884180

[B66] Nomura T. Hattori M. Osumi N. (2009). Reelin, radial fibers and cortical evolution: insights from comparative analysis of the mammalian and avian telencephalon. Dev. Growth Differ. 51, 287–297. 10.1111/j.1440-169X.2008.01073.x 19210541

[B67] Nomura T. Murakami Y. Gotoh H. Ono K. (2014). Reconstruction of ancestral brains: exploring the evolutionary process of encephalization in amniotes. Neurosci. Res. 86, 25–36. 10.1016/j.neures.2014.03.004 24671134

[B68] Nomura T. Takahashi M. Hara Y. Osumi N. (2008). Patterns of neurogenesis and amplitude of Reelin expression are essential for making a mammalian-type cortex. PLoS ONE 3:e1454. 10.1371/journal.pone.0001454 18197264 PMC2175532

[B69] Ohkubo Y. Uchida A. O. Shin D. Partanen J. Vaccarino F. M. (2004). Fibroblast growth factor receptor 1 is required for the proliferation of hippocampal progenitor cells and for hippocampal growth in mouse. J. Neurosci. 24, 6057–6069. 10.1523/JNEUROSCI.1140-04.2004 15240797 PMC6729672

[B70] O'Leary D. D. M. Chou S.-J. Sahara S. (2007). Area patterning of the mammalian cortex. Neuron 56, 252–269. 10.1016/j.neuron.2007.10.010 17964244

[B71] O'Leary D. D. Sahara S. (2008). Genetic regulation of arealization of the neocortex. Curr. Opin. Neurobiol. 18, 90–100. 10.1016/j.conb.2008.05.011 18524571 PMC2677555

[B72] Parent A. Olivier A. (1970). Comparative histochemical study of the corpus striatum. J. Hirnforsch. 12, 73–81. 5499697

[B73] Piñon M. C. Tuoc T. C. Ashery-Padan R. Molnár Z. Stoykova A. (2008). Altered molecular regionalization and normal thalamocortical connections in cortex-specific Pax6 knock-out mice. J. Neurosci. 28, 8724–8734. 10.1523/JNEUROSCI.2565-08.2008 18753373 PMC3844775

[B74] Poluch S. Juliano S. L. (2015). Fine-tuning of neurogenesis is essential for the evolutionary expansion of the cerebral cortex. Cereb. Cortex 25, 346–364. 10.1093/cercor/bht232 23968831 PMC4351427

[B75] Pownall M. E. Isaacs H. V. (2010). FGF Signalling in Vertebrate Development. San Rafael, CA: Morgan & Claypool Life Sciences. 21452439

[B76] Puelles L. (2001). Thoughts on the development, structure and evolution of the mammalian and avian telencephalic pallium. Philos. Trans. R. Soc. Lond. B Biol. Sci. 356, 1583–1598. 10.1098/rstb.2001.0973 11604125 PMC1088538

[B77] Puelles L. Kuwana E. Puelles E. Bulfone A. Shimamura K. Keleher J.. (2000). Pallial and subpallial derivatives in the embryonic chick and mouse telencephalon, traced by the expression of the genes Dlx-2, Emx-1, Nkx-2.1, Pax-6, and Tbr-1. J. Comp. Neurol. 424, 409–438. 10.1002/1096-9861(20000828)424:3<409::AID-CNE3>3.0.CO;2-7 10906711

[B78] Rakic P. (2009). Evolution of the neocortex: a perspective from developmental biology. Nat. Rev. Neurosci. 10, 724–735. 10.1038/nrn2719 19763105 PMC2913577

[B79] Reep R. L. Finlay B. L. Darlington R. B. (2007). The limbic system in Mammalian brain evolution. Brain Behav. Evol. 70, 57–70. 10.1159/000101491 17409735

[B80] Reiner A. (1991). A comparison of neurotransmitter-specific and neuropeptide specific neuronal cell types present in the dorsal cortex of reptiles with those present in the isocortex of mammals. Brain Behav. Evol. 38, 53–91. 1683805 10.1159/000114379

[B81] Rowe T. B. Shepherd G. M. (2015). The role of ortho-retronasal olfaction in mammalian cortical evolution. J. Comp. Neurol. [Epub ahead of print]. 10.1002/cne.23802 25975561 PMC4898483

[B82] Roy A. Gonzalez-Gomez M. Pierani A. Meyer G. Tole S. (2014). Lhx2 regulates the development of the forebrain hem system. Cereb. Cortex 24, 1361–1372. 10.1093/cercor/bhs421 23307637 PMC3977624

[B83] Rubenstein J. L. (2011). Annual research review: development of the cerebral cortex: implications for neurodevelopmental disorders. J. Child Psychol. Psychiatry 52, 339–355. 10.1111/j.1469-7610.2010.02307.x 20735793 PMC3429600

[B84] Sansom S. N. Griffiths D. S. Faedo A. Kleinjan D. J. Ruan Y. Smith J.. (2009). The level of the transcription factor Pax6 is essential for controlling the balance between neural stem cell self-renewal and neurogenesis. PLoS Genet. 5:e1000511. 10.1371/journal.pgen.1000511 19521500 PMC2686252

[B85] Schlosser G. (2005). Evolutionary origins of vertebrate placodes: insights from developmental studies and from comparisons with other deuterostomes. J. Exp. Zool. B Mol. Dev. Evol. 304, 347–399. 10.1002/jez.b.21055 16003766

[B86] Shanmugalingam S. Houart C. Picker A. Reifers F. Macdonald R. Barth A.. (2000). Ace/Fgf8 is required for forebrain commissure formation and patterning of the telencephalon. Development 127, 2549–2561. 10821754 10.1242/dev.127.12.2549

[B87] Shimamura K. Rubenstein J. (1997). Inductive interactions direct early regionalization of the mouse forebrain. Development 124, 2709–2718. 9226442 10.1242/dev.124.14.2709

[B88] Shimogori T. Banuchi V. Ng H. Y. Strauss J. B. Grove E. A. (2004). Embryonic signaling centers expressing BMP, WNT and FGF proteins interact to pattern the cerebral cortex. Development 131, 5639–5647. 10.1242/dev.01428 15509764

[B89] Striedter G. F. (1997). The telencephalon of tetrapods in evolution. Brain Behav. Evol. 49, 179–213. 10.1159/000112991 9096908

[B90] Striedter G. F. (2015). Evolution of the hippocampus in reptiles and birds. J. Comp. Neurol. [Epub ahead of print]. 10.1002/cne.23803 25982694

[B91] Striedter G. F. Northcutt R. G. (1991). Biological hierarchies and the concept of homology. Brain Behav. Evol. 38, 177–189. 10.1159/000114387 1663811

[B92] Suárez R. Gobius I. Richards L. J. (2014). Evolution and development of interhemispheric connections in the vertebrate forebrain. Front. Hum. Neurosci. 8:497. 10.3389/fnhum.2014.00497 25071525 PMC4094842

[B93] Subramanian L. Remedios R. Shetty A. Tole S. (2009). Signals from the edges: the cortical hem and antihem in telencephalic development. Semin. Cell Dev. Biol. 20, 712–718. 10.1016/j.semcdb.2009.04.001 19446478 PMC2791850

[B94] Sugahara F. Aota S. I. Kuraku S. Murakami Y. Takio-Ogawa Y. Hirano S.. (2011). Involvement of Hedgehog and FGF signalling in the lamprey telencephalon: evolution of regionalization and dorsoventral patterning of the vertebrate forebrain. Development 138, 1217–1226. 10.1242/dev.x059360 21343370

[B95] Sur M. Rubenstein J. L. R. (2005). Patterning and plasticity of the cerebral cortex. Science 310, 805–810. 10.1126/science.1112070 16272112

[B96] Suzuki I. K. Kawasaki T. Gojobori T. Hirata T. (2012). The temporal sequence of the mammalian neocortical neurogenetic program drives mediolateral pattern in the chick pallium. Dev. Cell 22, 863–870. 10.1016/j.devcel.2012.01.004 22424929

[B97] Tarabykin V. Stoykova A. Usman N. Gruss P. (2001). Cortical upper layer neurons derive from the subventricular zone as indicated by Svet1 gene expression. Development 128, 1983–1993. 10.1038/ng1096-218 11493521

[B98] Toyoda R. Assimacopoulos S. Wilcoxon J. Taylor A. Feldman P. Suzuki-Hirano A.. (2010). FGF8 acts as a classic diffusible morphogen to pattern the neocortex. Development 137, 3439–3448. 10.1242/dev.055392 20843859 PMC2947756

[B99] Tuoc T. C. Radyushkin K. Tonchev A. B. Piñon M. C. Ashery-Padan R. Molnár Z.. (2009). Selective cortical layering abnormalities and behavioral deficits in cortex-specific Pax6 knock-out mice. J. Neurosci. 29, 8335–8349. 10.1523/JNEUROSCI.5669-08.2009 19571125 PMC6665651

[B100] Vasistha N. A. García-Moreno F. Arora S. Cheung A. F. P. Arnold S. J. Robertson E. J.. (2014). Cortical and clonal contribution of Tbr2 expressing progenitors in the developing mouse brain. Cereb. Cortex 25, 3290–3302. 10.1093/cercor/bhu125 24927931 PMC4585488

[B101] Vied C. M. Freudenberg F. Wang Y. Raposo A. A. S. F. Feng D. Nowakowski R. S. (2014). A multi-resource data integration approach: identification of candidate genes regulating cell proliferation during neocortical development. Front. Neurosci. 8:257. 10.3389/fnins.2014.00257 25191221 PMC4139594

[B102] Wang Y. Brzozowska-Prechtl A. Karten H. J. (2010). Laminar and columnar auditory cortex in avian brain. Proc. Natl. Acad. Sci. U.S.A. 107, 12676–12681. 10.1073/pnas.1006645107 20616034 PMC2906560

[B103] Ypsilanti A. R. Rubenstein J. L. (2015). Transcriptional and epigenetic mechanisms of early cortical development: An examination of how Pax6 coordinates cortical development. J. Comp. Neurol. [Epub ahead of print]. 10.1002/cne.23866 26304102 PMC4706819

[B104] Zhou C. J. Borello U. Rubenstein J. L. R. Pleasure S. J. (2006). Neuronal production and precursor proliferation defects in the neocortex of mice with loss of function in the canonical Wnt signaling pathway. Neuroscience 142, 1119–1131. 10.1016/j.neuroscience.2006.07.007 16920270

